# Recent Study of Separation and Identification of Micro- and Nanoplastics for Aquatic Products

**DOI:** 10.3390/polym15214207

**Published:** 2023-10-24

**Authors:** Jin Xu, Gan Wu, Hao Wang, Zhaoyang Ding, Jing Xie

**Affiliations:** 1 College of Food Science and Technology, Shanghai Ocean University, Shanghai 201306, China; m220351095@st.shou.edu.cn (J.X.); m210300839@st.shou.edu.cn (G.W.); 2 National Pathogen Collection Center for Aquatic Animals, Shanghai Ocean University, No. 999, Huchenghuan Road, Shanghai 201306, China; h-wang@shou.edu.cn; 3 Marine Biomedical Science and Technology Innovation Platform of Lin-gang Special Area, Shanghai 201306, China; 4 Shanghai Engineering Research Center of Aquatic-Product Processing & Preservation, Shanghai 201306, China

**Keywords:** micro- and nanoplastics, aquatic products, food safety, environment, separation, identification

## Abstract

Micro- and nanoplastics (MNPs) are polymeric compounds widely used in industry and daily life. Although contamination of aquatic products with MNPs exists, most current research on MNPs focuses on environmental, ecological, and toxicological studies, with less on food safety. Currently, the extent to which aquatic products are affected depends primarily on the physical and chemical properties of the consumed MNPs and the content of MNPs. This review presents new findings on the occurrence of MNPs in aquatic products in light of their properties, carrier effects, chemical effects, seasonality, spatiality, and differences in their location within organisms. The latest studies have been summarized for separation and identification of MNPs for aquatic products as well as their physical and chemical properties in aquatic products using fish, bivalves, and crustaceans as models from a food safety perspective. Also, the shortcomings of safety studies are reviewed, and guidance is provided for future research directions. Finally, gaps in current knowledge on MNPs are also emphasized.

## 1. Introduction

Due to a well-developed economy and demand from people, a large amount of plastic pollution is produced every year [1]. Some of these plastics are at the nanoscale and not easily detected, and more and more plastics are broken down into tiny particles by external factors when they enter the aquatic environment. These plastics exhibit a significant surface area, often acting as carriers for a wide array of pollutants. Apart from organic pollutants, they also adsorb inorganic contaminants such as heavy metals and microbial pollutants in the form of biofilms.

In industrial production, social activities, and daily home life, plastic products and various types of plastic-containing wastes are generated and infiltrate aquatic environments through methods like direct discharge, intentional dumping, wind-driven transport, rainfall erosion, fishing and shipping activities, sewage discharges, and drifting movements [2,3,4,5]. During this process, plastics gradually degrade into tiny particles known as micro- and nanoplastics (MNPs). MNPs can be categorized as primary or secondary MNPs [6], Primary MNPs refer to plastic fragments or particles that are less than or equal to 5 mm in size, including fiber and other plastic particles originating from clothing [7]. Secondary MNPs are derived from the degradation of larger plastic products in the environment, such as water and soda bottles, fishing nets, and plastic bags [8]. MNPs are chemically stable and decompose slowly, persisting in aquatic environments, through the food chain and ingestion, and may enter aquatic products to pose a potential toxicity risk to aquatic products. Human exposure to MNPs is primarily through ingestion, inhalation, and skin contact [9]. In general, MNP contamination in aquatic products is considered as a vital source in the ingestion route. However, current research on MNPs is focused on environmental [10], ecological [11], and some toxicological studies [12]. Few studies on aquatic products have been undertaken in terms of food safety.

Aquatic products constitute a substantial proportion of animal-based consumption worldwide, yielding significant economic benefits to coastal areas. However, the widespread presence of MNPs in aquatic ecosystems and aquaculture systems has resulted in diverse detrimental effects on both human health and aquatic organisms. Moreover, MNPs exhibit seasonal and spatial variations, resulting in differing concentrations within the same water bodies at different times [13]. MPs in aquatic products vary depending on the waters and sources. The most commonly used plastic materials in daily life are HDPE, LDPE, PP, PVC, and PET [14]. Moreover, the presence of MNPs exhibits both seasonal and spatial variations, leading to varying levels within the same waters at different times [13]. In mussels captured from the Mediterranean, polymers accounted for eighty-four percent of the MNPs found, with synthetic cellulose being the second most prevalent. Similarly, suspension-cultured and bottom-cultured oysters from the same farms showed varying concentrations of MNPs [15,16].

Given the prevalence of MNPs in aquatic organisms, it has become critical to study the separation and identification of MNPs in aquatic products. Nowadays, researchers employ diverse methods to separate and detect MNPs by investigating their physical and chemical properties. Techniques such as microscopic counting and scanning electron microscopy (SEM) are frequently utilized to detect MNPs as they enable direct observation and recording of the morphology, size, and structure of MNPs without the necessity for chemical treatment of the samples [17]. MNPs typically consist of specific chemical compositions, which enable researchers to detect and identify MNPs through techniques such as Raman spectroscopy, pyrolysis-gas chromatography/mass spectrometry (Py-GC/MS), and high-performance liquid chromatography (HPLC) [18,19].

In an effort to reduce the interference of organic matter in organisms, Claessens et al. employed chemical ablation for the first time to extract MNPs from aquatic organisms [20]. However, the lack of a standardized extraction method and the use of different materials and pore sizes of filter membranes during the separation process can lead to varying detection rates of MNPs [21]. The harmonization of practical methods for the extraction and isolation of MNPs from aquatic organisms is currently a critical scientific challenge in MNP research. The lack of reliable data on the concentration, particle size distribution, and polymer composition of MNPs in aquatic organisms is also attributable to inadequate operational procedures for their extraction, separation, and identification. Results from different studies are not comparable or even conclusive. The inconsistency of MNP analytical methods not only impairs related scientific research, but also hampers the ability to analyze MNPs. This deficiency hinders the comprehensive assessment of MNP toxicity [13]. Additionally, determining the exposure pathways and dosages of MNPs remains challenging, and the assessment of health effects is significantly influenced by factors such as the pathway of exposure, the amount inhaled or ingested, and the limited representativeness of current collection methods. Presently, only studies investigating the toxicity of individual chemical substances in a single medium can be conducted, making it impossible to evaluate the combined effects of MNPs. Another critical issue is the lack of a standardized evaluation system. Universally applicable standards for the health and environmental hazard assessment of MNPs are currently absent [2], posing difficulties in data comparison and potentially leading to errors.

In this review, we summarize existing peer-reviewed articles on MNPs in aquatic products. We also discuss the number and types of MNPs present in aquatic products, their physical and chemical properties, and the latest analytical methods for isolating and characterizing them. The advantages and disadvantages of the various analytical methods are compared, and research requirements for improving the assessment of aquatic products exposure to MNPs through food consumption are presented. Challenges to the development of standardized methods for MNPs are analyzed, and gaps in current food safety research are identified. The necessity and urgency of establishing standardized analytical methods for MNPs in aquatic organisms are emphasized.

## 2. Current Situation of MNP Contamination in Aquatic Products

**Figure 1:**
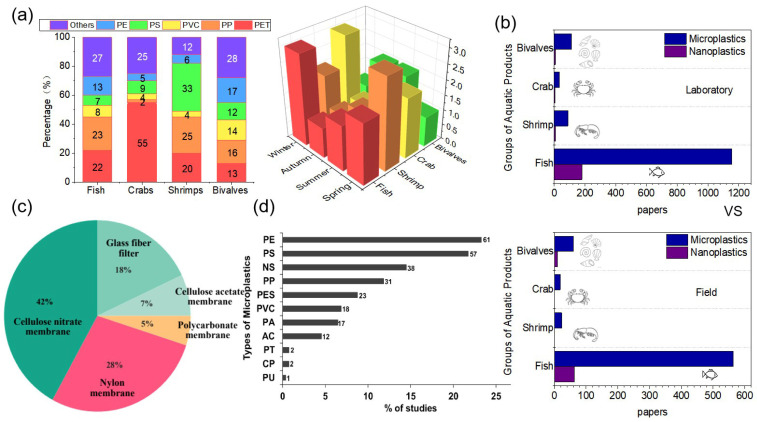
(**a**) The proportion of major MNPs species in fish, shrimp, crab, and bivalves; the proportion of the same MNPs measured in fish, shrimp, crab, and bivalves in different seasons [22,23,24,25,26,27,28,29,30,31,32,33,34]. (**b**) Field and laboratory exposures of aquatic products in the study of the ingestion and effects of microplastics and nanoplastics. (**c**) Broad categories of MNPs commonly found in aquatic environmental food webs. (**d**) Percentage of studies categorized by types of microplastics in aquatic products (reprinted from ref. [35]). Note: Crustaceans in this review are categorized as shrimp and crab.

MNPs are widely present in the aquatic environment, raising concerns about their presence in commercially important seafood, having become an emerging food safety issue. The investigation of MNPs in aquatic products remains insufficient, leaving potential gaps in our understanding. Furthermore, MNPs could adsorb harmful chemicals that can accumulate in the human body, posing significant health risks. The variation in MNPs in different aquatic products as well as the fluctuating content of the same MNPs in different aquatic products during various seasons (as shown in Figure 1) highlight the complexity of the issue.

Comparatively higher individual detection rates of MNPs were found in fish as opposed to other aquatic products, with fish showing a significantly greater abundance of solitary MNP particles. The types of MNPs isolated from these aquatic products include various forms of fiber, fragments, granules, and sheets. Fiber was the most widespread shape in MNPs, accounting for over 70% of the entities examined. These fiber MNPs mainly consisted of artificial filaments constituting a semi-synthetic polymer, comprising nearly half (48.92%) of all detected plastic polymers [36]. Therefore, it is even more important to understand the recent presence of MNPs in current aquatic products before summarizing the separation and identification of MNPs (Table 1).

### 2.1. Occurrence of MNPs in Aquatic Products

#### 2.1.1. Fish

Before quantifying the quantity of MNPs present, it is crucial to acknowledge that the levels of MNPs significantly vary across different water sources and regions. For instance, Catarino’s study reported that only 1.80% of the 15 samples derived from studying 4389 species contained detectable plastic particles. Even among 400 randomly sampled fish, a mere of two plastic were observed. However, populations heavily reliant on fish resources or consuming fish from areas experiencing elevated plastic contamination may face higher exposures [6]. The abundance of MNPs in the stomach and intestine of cultured hybrid groupers from the Pearl River Estuary was detected at 35.36 particles per individual or 0.62 particles per gram. The number of MNPs in the intestine of the fish (23.91 particles per individual or 1.10 particles per gram) was higher than in the stomach (12.80 particles per individual or 0.37 particles per gram) [37]. According to a survey, the levels of MNPs in farmed aquatic organisms growing in the lake were found to be higher compared to other water sources [43]. Yuan et al. investigated MNP contamination in 349 sea bass specimens from coastal areas of Jiangsu Province, China. The abundance of MNPs in sea bass individuals measuring less than 1 mm was determined to be 1.03 ± 1.04 MNPs, and the abundance of MNPs in fish farmed in lake is generally higher than in fish farmed in the sea [14]. The level of MNP contamination in sea bass from the Jiangsu coast was relatively low to moderate compared to other regions of China. The reason for this disparity is attributed to urban sewage discharge and household waste, which contribute to increased levels of MNPs in the lake. Consequently, aquatic organisms raised on farms that utilize the lake as a water source become enriched in enriched with MNPs in their bodies through the food chain, leading to the observed higher levels of MNPs in these organisms. To explore the abundance of MNPs in pelagic edible fish in India, Daniel et al. sampled 270 fish, resulting in 41.10% of the fish containing MNPs in edible tissue, with an average abundance of 0.07 ± 0.26 MNPs per fish [38]. Such low detection rates may indicate potentially low levels of exposure to humans.

#### 2.1.2. Bivalves

Viable bivalves, including raw oysters and steamed clams, have become a popular choice for seafood lovers and are no longer restricted to coastal areas. Thanks to the rapid development of aquaculture and improvements in seafood preservation and transportation, bivalves are now widely available. However, with the increasing pollution caused by MNPs, especially in coastal aquaculture ecosystems, the accumulation of MNPs in bivalve organisms has become a significant problem. MNPs have been detected in many coastal bivalve species [68]. Despite the presence of MNPs in bivalves, there are still important knowledge gaps regarding the influence of bio-morphology on the mechanisms of MNPs accumulation. Additionally, the potential impact of seasonality on changes in the levels of MNPs in bivalves remains poorly studied. These gaps in understanding could hinder the accurate identification of risks related to food safety in bivalves. Fiber was the most common shape of MNPs, accounting for more than half of the total MNPs in eight of the species. In a study by Joshy et al. on bivalves from the Paraiyar River in India, MNPs were observed in the digestive glands (22.8 particles g^−1^) and gills (29.6 particles g^−1^) [69]. Britta R. et al. quantified Pacific oysters and Pacific razor clams from 15 coasts in Oregon, USA, and found that both oysters and razor clams contained MNPs, with the majority (over 99%) being fiber. On average, whole oysters contained 10.95 ± 0.77 MNP particles, while razor clams contained 8.84 ± 0.45 MNP particles [70]. Routine testing in Chinese coastal watersheds revealed higher levels of MNPs in spring samples compared to summer oyster samples, suggesting that MNP contamination in bivalves varies seasonally, with bivalves potentially being safer in the summer [71]. Overall, the results indicate that MNP contamination is widespread in bivalves, with fiber being the most prevalent shape of MNPs found in bivalve tissue at each site. The size of MNPs smaller than 2 mm was also commonly observed. High levels of MNPs were detected in commercial bivalves from China and other regions.

#### 2.1.3. Crustaceans

Decapod crustaceans belong to the phylum Arthropoda and are the most abundant animals on the planet in terms of biomass. Some decapod crustaceans hold great significance as seafood products, and they are highly valued in commercial fisheries and aquaculture worldwide. Crustaceans are also utilized in various ways, including as food, animal feed, and for chitin extraction. The contamination of local marine ecosystems by MNPs is becoming an increasingly concerning environmental issue. Such contamination can potentially have adverse effects on the safety of seafood when consumed by marine creatures. In general, the levels of MNPs detected in crustaceans were found to be higher (ranging from 26.4% to 85.40%) compared to fish (ranging from 37.60% to nearly 67%). For instance, in Indian white shrimp, the levels of MNPs ranged from 0.69 ± 0.48 particles per gram to 3.45 ± 0.04 particles per gram [44]. Moreover, apart from the two prevalent plastics, polypropylene and polyethylene, cellophane and synthetic fiber were also discovered in the samples of shrimp fishery goods [45]. This indicates that a variety of plastic materials are present in the marine environment and can be ingested by marine organisms such as shrimp, further highlighting the potential risks of MNP contamination in aquatic products. In a study of aquatic shrimp products from a highly productive farming site in China, Wu et al. identified the presence of cellulose, polyamide, acrylonitrile, and polyethylene in aquatic shrimp products. Among these, polypropylene and polyethylene terephthalate cellulose were the predominant polymers, accounting for 67% to 84% of cultured shrimp, with 0.95 to 2.10 items per individual [29]. Interestingly, the investigation did not reveal any regional trend in plastic consumption by the shrimp, but there were temporal variations. MNPs consumption was significantly increased in October compared to March. This increase in MNPs consumption in October could be attributed to the fact that the shrimps were more active during this time, building up fat reserves in preparation for winter. Crab products are now sourced not only from marine capture but also from aquaculture systems. However, aquaculture ecosystems face serious threats from various pollutants, including MNPs. Crab ponds have been found to have higher levels of MNPs compared to fishponds and nearby natural lakes. In these ponds, debris and fiber are the primary shapes of MNPs, and the presence of smaller MNPs is positively correlated with the proportion of debris MNPs [55]. Xiong et al. conducted a study in the central freshwater aquaculture area of Hong Hu, China, and found MNPs in both crab breeding ponds and nearby natural lakes [56]. Similarly, Xu collected a total of 38 species of bivalves and crabs in the Hong Kong area and observed a mean number of MNPs ranging from 0 to 9.68 grains per gram or 0 to 18.4 grains per gram. Approximately 26% of the suspected MNPs were confirmed to be synthetic polymers, including CP, PET, and PA [57].

### 2.2. Hazards to Aquatic Products and Human Health

The small size of MNPs enables them to translocate through biofilms, affecting aquatic organisms through a mechanism similar to endocytosis, which can result in health issues for aquatic products [72]. Recent research indicates that MNPs can reach various organs in aquatic organisms after ingestion, leading to organ damage. While the exact impact of MNPs on human health is yet to be fully determined, it is crucial to consider the risk to humans from both the MNPs themselves and the toxic substances they may adsorb. Such considerations should be based on rigorous scientific research and account for factors such as dose and cumulative exposure. In order to provide a comprehensive understanding of the health implications from a food perspective, our research also incorporates studies pertaining to human health (Table 2).

### 2.3. Challenges in Investigating the Hazards of MNPs from a Food Perspective

While there is a growing body of research on the effects of MNPs on aquatic biota, some impact studies may be biased toward specific shapes of polymers, neglecting the reported occurrence, estimated releases to the environment, and bioavailability of MNPs in organisms and the environment. This potential bias should also be considered when using model organisms in laboratory assays. The trophic transfer of MNP particles and their associated toxins in aquatic food webs, as well as the potential health risks to human health resulting from exposure to MNPs through the ingestion of aquatic food, remain largely unknown. Given that it is widely recognized that NPs tend to be more toxic than MPs, it is expected that NPs would have similar toxic effects on the reproductive and nervous systems. In addition, studies on human lung epithelial cells suggest that NPs may also induce cell death. The size of the particles may often play a significant role, as NPs can diffuse through membranes that MPs cannot penetrate. For example, in laboratory experiments, zooplankton carrying NPs were fed to fish, and the results demonstrated that NPs were transferred to the fish and could cross the blood–brain barrier, causing brain damage and behavioral impairment in the fish. Considering the differences in habits and physiology between marine and freshwater species, it remains uncertain to what extent the findings from marine biology-based studies can be applied to freshwater species, and vice versa. However, research on NPs is relatively limited, leading most researchers to study them as equivalent to MNPs. This emphasizes the need for studies on the separation and identification of MNPs in order to gain a more comprehensive understanding of the impact of MNPs on aquatic products and human health.

## 3. Separation and Identification of MNPs in Aquatic Products

**Figure 2:**
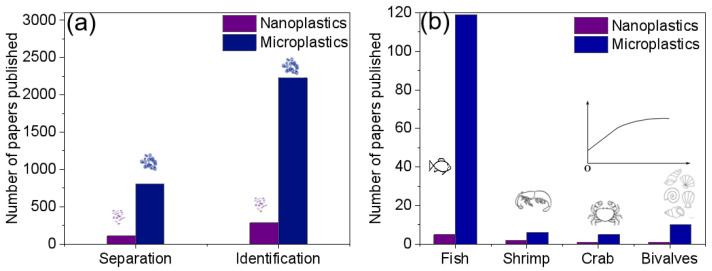
Shows the number of papers in subcategories of: (**a**) separation and Identification [nanoplastics and microplastics]; (**b**) aquatic products [microplastics and fish/shrimp/crab/bivalves, or nanoplastics and fish/shrimp/crab/bivalves].

Over the past decade, there has been a notable surge in research focused on separation and identification of MNPs in aquatic products, as evident in Figure 2. Despite the increasing number of publications on the subject, there remains a significant gap, especially concerning the measurement of NPs. We conducted topic searches on the Web of Science to gauge the current research status of MNPs. However, it is essential to note that there may be some overlap and subsets among the papers, making these statistics serve as rough estimates.

### 3.1. Separation Methods 

#### 3.1.1. MNPs Extraction

**Figure 3:**
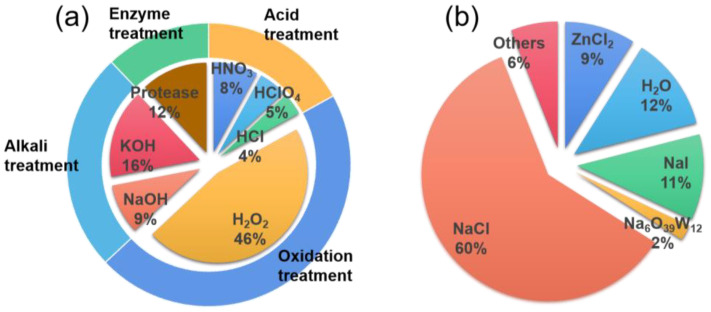
(**a**) The extraction methods of MNPs from aquatic products; (**b**) densitometric separation methods of MNPs from the biological organization of aquatic products.

Unlike the separation of MNPs from the environment and sediments, the extraction of MNPs from aquatic products necessitates an initial extraction procedure. This process is designed to mitigate the interference of organic matter and gain MNP particles suitable for subsequent analysis and identification. Figure 3a enumerates the current techniques employed for MNPs extraction from aquatic products.

Alkalis can disintegrate aquatic tissues by hydrolyzing chemical bonds and denaturing proteins. Currently, one of the most common methods to ablate MPs extracted from aquatic organisms is using 10% KOH. Ding et al. effectively used 10% KOH ablation of the esophagus, stomach, and intestine of fish at 60 °C for 24 h [86]. Dehaut et al. used the same method to treat MNPs quickly and effectively in aquatic products [87]. Still, silica-like substances present in the samples could not be decomposed by KOH, and the alkaline solution also caused severe degradation of polymers, such as PET and PVC.

Hydrogen peroxide (H_2_O_2_) is an effective oxidant for removing organic and biological matter. Thirty percent of H_2_O_2_ can make MPs smaller, thinner, and more transparent, facilitating the extraction of MNPs from aquatic samples. It is also effective in ablating the soft tissues of various shellfish, such as mussels, oysters, arks, and mud crabs [32,88]. Sodium hypochlorite (NaClO) is also suitable for removing organic debris from aquatic tissues. Monteiro et al. demonstrated that aquatic tissues can be effectively ablated with NaClO at room temperature, resulting in desirable digestion of the tissues [89]. However, the oxidative nature of H_2_O_2_ can cause discoloration of many polymers, as well as slight degradation of PP and PE. In this scenario, the NOAA recommends employing a Fenton reagent (H_2_O_2_/Fe^2+^), heating it to ablate pollutants in the sample [25].

Acids rapidly dissolve aquatic tissues by breaking down organic matter such as proteins, carbohydrates, and oils. Appropriately proportioned concentrations of HNO_3_ and HClO4 are generally used to dissolve the soft tissues of mussels. The tissues are first digested at room temperature and then completely dissolved by heating to boiling [90]. While HNO_3_ is excellent in dissolving PE, PS, and PET, it creates MNPs yellowing and inefficiency when dissolving other organics. Other research has discovered that, while HNO_3_ is successful at removing organics, oil and tissue residues remain, which may alter the final measurement of MPs [91,92].

Enzymatic digestion of aquatic tissues mainly involves the hydrolysis of proteins, although it is a less commonly used method. Cole et al. digested 0.20 g of planktonic samples with 500 g L^−1^ protease, and the digestibility was 90% at 50 °C for 2 h [93]. Courtene-Jones et al. used a range of proteolytic digestive enzymes, including trypsin, papain, and 250 collagenases, to develop the optimum digestion efficacy of mussel soft tissues. Among the enzymes tested, trypsin yielded the highest digestion efficiency (88%) after incubation at 40 °C for 30 min, and enzyme treatment had no detrimental effects on the investigated MNPs [94]. These enzymes, however, function poorly on dense organic materials and are substantially more expensive than inorganic oxidants, acids, and/or bases [92]. Consequently, a more effective enzymatic technique validation is required.

#### 3.1.2. MNPs Separation

MNPs are ubiquitous in aquatic ecosystems and potentially harmful in food sources. Given the prevalence of MNPs in aquatic products, it is imperative to study and compare their abundance in different species. Density-based techniques have emerged as a practical approach to extract and isolate MNPs from aquatic organisms. In order to provide the reader with a clear understanding of the shapes of reagents currently used for MNPs densitometric extraction, we have listed the most commonly used reagents in Figure 3b. In contrast, emerging techniques like electrostatic and magnetic separation present advantages, including cost-effectiveness, rapidity, and environmental friendliness, albeit accompanied by demanding instrumentation requirements. Although the distinctions among these methods, separation efficiency remains intricately linked to the properties of the MNPs, encompassing factors such as polymer composition, size, and morphology.

Based on the prevalent MNPs shape (Table 1) and their varying densities in different aqueous products, separation is achieved through distinct flotation techniques employing different media. MNPs with lower densities can be readily separated using water, while salt-saturated separation methods are employed for high-density MNPs shape. If the digested solution contains many undigested inorganic particles, density separation can be used to separate the MNPs. It is worth noting that not all salt saturated solutions are fit for extraction of high-density MNPs types. Currently, many researchers are using saturated sodium chloride solutions to isolate MNPs, in addition to zinc chloride and sodium iodide solutions are also more commonly used salt saturated solutions. Of course, some researchers have also tried to use substances such as potassium formate and oil to separate MNPs [95,96,97]. Additionally, thermal filtration of aquatic samples directly after digestion can expedite the filtration process. The choice of the membrane is crucial for the filtration and identification processes, and currently, cellulose or nylon membranes are dominant as they do not react with the solvent [98]. However, the lack of a uniform method of extraction and the use of different materials and pore sizes of filter membranes in the separation process can lead to varying detection rates of MNPs.

Field-Flow Fractionation (FFF) serves as a valuable separation technique widely applied for size and molar mass fractionation across a range of substances, including biopolymers, proteins, polymers, and nanoparticles. The principle behind this method entails the flow of a fluid suspension through a slender FFF channel while subjecting it to a perpendicular force field. In the research of Correia et al., this method is employed to separate MNPs found within Danish fish samples. This approach not only enables the effective isolation of MNPs from digested fish but also affords precise size determination [99]. 

With a number of magnetic materials being developed in recent years, magnetic separation offers a fast and low-cost solution for the removal of MNPs. Magnetic separation is mainly based on the hydrophobicity of MNPs, which allows them to be magnetized and bound to magnetic materials [100]. The movement of MNPs can be controlled by a magnet or by applying a magnetic field, this makes it easy to separate MNPs from water. Electrostatic separation enables the direct isolation of non-conductive MNPs from conductive media, facilitating the recovery of separated MNPs [101]. Conversely, oil separation emerges as a novel, cost-effective approach with high removal rates and minimal associated risks [102]. The lipophilic nature of MNPs allows them to migrate from water into the oil phase, irrespective of density differences. However, it is important to note that while oil separation boasts high removal efficiency, it may lack precision due to the potential adherence of contaminants from different aquatic sources to the surface of MNPs, thereby altering their lipophilicity.

### 3.2. Identification Methods

MNPs in aquatic products can be identified using various approaches, including optical, spectroscopic, and thermal methods. The graphical approach involves separating MNPs through filtration using microscopes or other tools, and then observing the filtered membrane with the naked eye or a microscope. MNPs are classified and counted based on their shape, size, and color on the membrane, and the results are further confirmed using instrumental methods [103]. However, the limitations of the visual method in terms of accuracy and polymer shape determination do not recommend it as a stand-alone extraction method. It should only be used as an aid for further analysis and identification of MNPs. To provide a clearer understanding of the currently available methods for detecting MNPs, and to aid researchers in selecting the most suitable detection method for their studies, this paper presents a summary and comparison of the advantages and disadvantages of various analytical techniques that have been used for MNP detection (Table 3).

Spectroscopy is mainly used to identify polymer compositions by obtaining information on the functional groups of MNPs [120]. Standard methods include Raman spectroscopy, Fourier infrared spectroscopy, and others. Fourier infrared spectroscopy (FT-IR) is widely used for the qualitative detection and compositional analysis of MNPs due to the advantages of non-destructive sample and piece pre-treatment [108]. After microscopically sorting aquatic samples from the coast by shape and size, researchers analyzed the composition of MNPs by FT-IR. They found that the main MNPs shape were fiber, polyethylene, and PET [59]. Surface micromorphology analysis of MNPs is an essential basis for studying their chemical properties. Currently, SEM-EDX is the most widely used method for surface micromorphology analysis of MNPs [34]. However, the detection of MNPs by a single method is susceptible to interference from false positive or false negative signals, resulting in low detection accuracy [121]. As a result, it is recommended that when employing this method to quantify MNPs, the aquatic products samples should be checked by microscope first and subsequently evaluated by spectroscopy. The visual method is integrated with the FT-IR form, in which MNPs are categorized by shape and color using the naked eye or a microscope, and then the kind of polymer is determined using FT-IR. TGA-FTIR-GC/MS is also a viable approach for analyzing the shape and total mass of MNPs and their additives in complicated samples; however, it does not allow for the counting of MNP particles [122].

**Figure 4:**
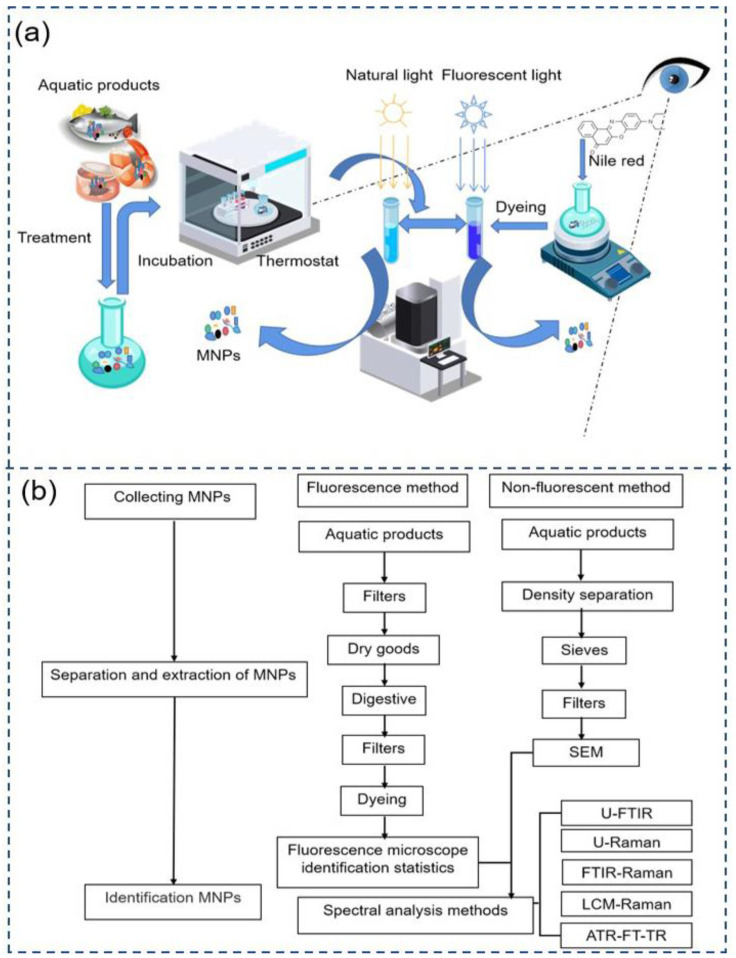
(**a**) Both a non-fluorescent technique and the advanced Nile red fluorescence staining technique for detecting MNPs (both based on SEM) were demonstrated; (**b**) classification of MNP detection methods.

Because MNPs in aquatic items are so small and difficult to recognize, a rapid and accurate micron or even nanoscale characterization of these MNPs is required. This challenge can only be overcome by continuously improving existing methods and developing new techniques for detecting and quantifying MNPs in aquatic samples, as well as continuously optimizing detection times and efficiency. In Figure 4, classification of detection methods by combining existing MNP detection methods, as well as a generalization and summary of fluorescence detection are presented.

Nile red staining-fluorescence spectroscopy/mass spectrometry, fluorescence properties of fluorescent whitening agents/visual examination, and Rose Bengal staining/visual inspection are generally well-established staining procedures for the analysis of MNPs [123]. Compared to the direct identification of MNPs by SEM, in the preprocessing session of MNPs, staining the MNPs not only eliminates interference at the separation and processing stage, but also reduces fluorescence errors in spectroscopic analysis. The most important thing is that after visual observation, spectroscopic and chromatographic analysis, or automated or semi-automated instrumental analysis combined with image analysis is performed, which can yield the physical and chemical characteristics of the MNPs. In the future, this could be a crucial area of study for the precise and quick detection of MNPs. Prata et al. stained the MNPs with Nile red after removing the staining residue with water and acetone in KOH digestion [106]. Under fluorescence microscopy, it was demonstrated that the MNPs were not significantly damaged and that the MNP particles were able to be well defined. Guzman et al. conducted a comparative analysis and exposure assessment of MNPs in Manila clams of varying sizes from Korea, employing μ-FTIR and Nile red staining techniques, the investigation revealed a substantial presence of MNPs in Manila clams [59].

To facilitate the quantification and classification of MNPs, Shi et al. developed a rapid and precise deep learning method for the recognition and identification of MNPs. This approach addresses a significant challenge in the analysis of MNPs samples and data [124]. Although recent advances in automation have led to significant progress in the field of MNP research, most of these methods still require the use of expensive equipment, such as μ-FTIR analysis or μ-Raman spectroscopy [125], and they often require long computational times.

### 3.3. Physical and Chemical Properties of MNPs

Most scientific researchers commonly define MP particles as those ranging from 5 mm to 1 μm in diameter, while NPs are particles with a diameter of less than 1 μm. MPs can take on various shapes, which are classified as spheres, fiber, fragments, films, and particles. The sizes of MPs can fall into several categories, including <50 μm (including NPs), 50–100 μm, 100–200 μm, 200–400 μm, 400–800 μm, 800–1600 μm, >160 μm, or unspecified [13]. There is a widely accepted consensus that plastic materials are currently undergoing weathering and/or degradation, resulting in increasingly smaller pieces. This reduction in size is attributed to several factors, including UV radiation, chemical reactions, mechanical forces, and natural biological processes [16]. Pollutant adsorption has predominantly focused on persistent polycyclic aromatic hydrocarbons (PAHs), aromatic hydrocarbons, aliphatic hydrocarbons, chlorobenzenes (CBs), polychlorinated biphenyls (PCBs), and pesticides [126]. Many studies have already confirmed the adsorption of ionizable organic pollutants, like antibiotics, by MNPs. The main mechanisms of pollutant adsorption include hydrophobic interactions, partitioning, electrostatic interactions, and other non-covalent interactions, which usually work together in the adsorption process.

Both primary and secondary MPs can enter aquatic organisms and cause significant ecosystem damage [127]. MNPs are commonly found in aquatic products, and they can vary in size and color, ranging from large to small. Additionally, they exhibit different harmful effects on both humans and aquatic products. Moreover, MNPs can synergistically interact with other chemicals, posing a severe threat to aquatic life [128]. When organisms ingest MNPs, along with the chemicals they carry, these substances are distributed into the body. Consequently, they may accumulate in tissues with high lipid content or enter higher trophic levels (including humans) through the food web [129]. Due to their large specific surface area, MNPs can act as sorbent carriers for various pollutants, including PAHs, PCBs, polybrominated diphenyl ethers (PBDEs), organochlorine pesticides (OCPs), and heavy metal pollutants (e.g., zinc, lead, nickel, and cadmium), as well as antibiotics. This interaction modifies the environmental behavior and toxic effects of these organic pollutants [130]. PP primarily contain the elements C, O, Mg, Si, Br, Ca, Zn, Fe, and Al [131]. Additionally, PET, which also contain Cr, Zn, Pb, and Cd, were found to have small amounts of fat, protein, and carbohydrates adhered to their surfaces [132]. Moreover, the most common polymers used in aquaculture systems include PE, PS, PP, PVC, di (2-Ethylhexyl) phthalate (DEHP), and dibutyl phthalate (DBP) [133]. Perfluoroalkyl and polyfluoroalkyl (PFAS) are more likely to be adsorbed on PS. Due to the high heat resistance of PFAS, even complete cooking of contaminated food is ineffective in destroying it [133]. Aquatic organisms have been found to contain plasticizers, flame retardants, antioxidant stabilizers, and UV stabilizers, as these substances are used in aquaculture systems for necessary disinfection and water quality assurance [1]. Additionally, additives such as PBDEs, nonylphenols, BPA, and Triclosan are commonly used to enhance the performance of plastics [134]. If these substances enter biological matrices through the consumption of MNPs, they may have toxic effects.

Due to their chemical stability, toxic chemicals tend to adsorb onto MNPs rather than react with each other [135]. It has been observed that plastics with a highly crystalline structure exhibit reduced adsorption of organic chemicals compared to amorphous plastics. In a study by Zou et al., using PVC and CPE as polar model adsorbents (both containing chlorine (Cl)), and comparing them with polyethylene plastics HPE and LPE, which solely consist of C-C bonds (-C-C-) and methylene (-CH2-), despite having similar chemical structures, CPE and PVC displayed different affinities for metal adsorption [3]. This inconsistency suggests that surface properties, such as functional groups or charges of the plastics, play a crucial role in metal adsorption onto MNPs. Furthermore, a previous study conducted by Brennecke et al. discovered that the surface area in Cu^2±^ adsorbed PVC was significantly larger than that in PS, possibly due to the high surface area and/or polarity of PVC [136]. Consequently, the combined impacts of MNPs and associated pollutants on aquatic products is a topic of scientific interest due to these concerns.

### 3.4. Identification and Analysis Methods of MNPs Based on a Food Safety Perspective

Various exposure routes for MNPs, including inhalation, indirect ingestion, and direct ingestion through the food chain, have been identified as significant factors influencing their risk assessment. The concentration of MNPs, as well as their size, shape, and polymer shape, have all been recognized as important considerations. In the MNPs risk assessment techniques, both qualitative and quantitative analyses are commonly employed [137]. Several methods, such as SangKham’s hazard ranking models, ecological risk index methods, pollution load indices, and worst-case scenarios based on data from previous studies, are utilized [138]. Semi-quantitative risk assessment is another widely used approach, involving the scoring of risk factor categories, which are then arithmetically calculated to determine the final risk based on the severity of the hazard [139]. To gather data on MNP concentration, Demopoulos et al. developed a custom risk of bias (Rob) assessment tool that evaluates studies in four categories: design, sampling, analysis, and reporting [65]. The tool includes a checklist of questions covering all aspects of experimental protocol development, execution, and reporting, with studies being rated as high, low, or unclear, accompanied by a rationale for each rating. As most MNP particles in aquatic products are found in their digestive tracts, assessing exposure based on the abundance of MNPs in these tracts remains crucial.

**Figure 5:**
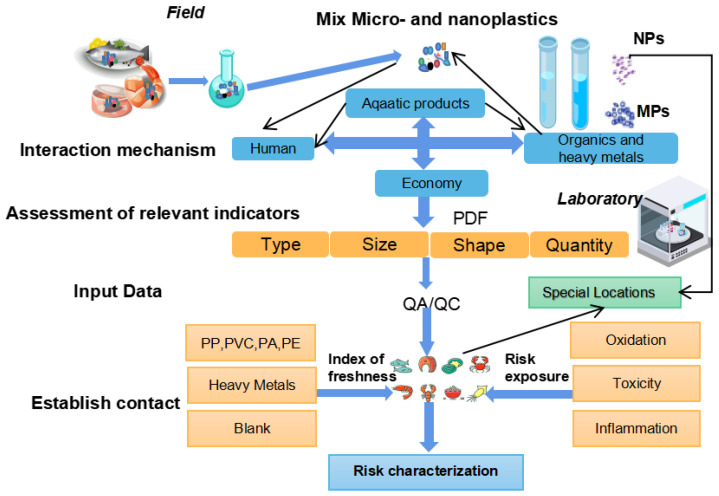
The framework for the identification and analysis of MNPs based on a food safety perspective.

We combined with the detection and evaluation of aquatic products from a food perspective by other researchers, have summarized a framework based on their findings (Figure 5). This visual representation outlines the diverse steps, methods, and criteria involved in assessing the presence and potential risks associated with MNPs in various aquatic food sources. The PDF is a mathematical function generated by fitting empirical data (size, shape, and density) from a large number of MNP particles in experiments [140], and when combined with FTIR measurements, it can be used to quantify certain properties of MNPs. The framework aims to avoid simplification with categories and focuses on characterizing toxicologically relevant particles using PDFs. It also incorporates quality assurance and quality control (QA/QC) screening methods to assess whether exposure and impact data are fit for purpose. Additionally, the framework links food safety to MNP toxicity experiments to enable a comprehensive risk assessment of MNPs from a food safety perspective. This approach provides a more nuanced and comprehensive understanding of the risks associated with MNPs in aquatic products, ensuring that assessments are robust and informative for decision-making processes.

### 3.5. Multiple Factors Contributing to the Uncertainty of the Effects of MNPs

Regarding NPs particles, the current understanding of the correlation between particle characteristics and toxicity primarily stems from laboratory studies conducted with artificially produced particles. Consequently, limited insight is available concerning the impact of natural NPs present in aquatic ecosystems and their effects on aquatic products. Credible trial and testing methods for identifying these particles beyond their physical attributes and some known toxic properties are lacking. Furthermore, differentiating between various MNPs proves challenging, complicating experimental evaluation.

The prevalent mass-based characterization technique has been widely used due to its convenience, but it has limitations in revealing the effects of actual exposure levels on low-toxicity or non-target species. To ensure accurate measurements of different shapes and sizes of MNPs, comprehensive equipment, methods, and high sample collection and processing standards are essential to avoid bias. However, there are several limitations in the measurement and characterization of MNPs in most papers. Firstly, imaging validation and uniform characterization methods are often lacking, leading to uncertainties about potential contamination by non-MNPs substances and raising concerns about errors in the risk assessments of MPs. Secondly, seasonal, and spatial fluctuations in MNPs abundance can result in inaccurate predictions if identical evaluation criteria are applied. The presence and abundance of MNPs in water products can vary significantly between field observations and laboratory characterizations and from one region to another. Furthermore, despite recent improvements in quality assessments, actual intake levels of MNPs may have been overestimated in some cases. Rigorous studies have shown that the number of particles in gastrointestinal tracts of organisms can be lower than previously reported. Assessing the potential health risks of MNPs is a complex task due to the vast number of combinations of polymer types, sizes, shapes, and chemical surface groups. This complexity makes it challenging to evaluate precise health risks associated with these particles within a given framework. While progress has been made in establishing the presence and characterization of MNPs in aquatic products, the task becomes even more difficult when dealing with NPs, as they present additional challenges in detection, characterization, and risk assessment. Therefore, continuous efforts and advancements are needed to improve our understanding of the risks posed by MNPs to both aquatic ecosystems and human health.

### 3.6. Challenges in Separation and Identification MNPs

Separating and identifying NPs in natural environments is significantly more difficult than in laboratory studies, primarily due to the presence of confounding factors, such as heavy metal contamination. Moreover, distinguishing NPs from MNPs can be a daunting task, as plastics at the nanoscale share similar characteristics with other particulate materials, leading to frequent confusion between the two. Therefore, establishing an efficient, comprehensive, and accurate detection method for MPs requires careful consideration of the distinct differences or commonalities that exist among various MNPs forms. Furthermore, particle number, rather than mass, is of greater importance in assessing the health effects of NPs. Relying solely on mass characterization techniques is insufficient for detecting them [141]. Since NPs are too small for light-based microscopy using UV, visible, or IR light alone, additional advanced approaches are necessary for effective identification and quantification of these particles. Finally, the chemical composition of NPs poses additional challenges in their characterization, as they predominantly consist of carbon, hydrogen, and oxygen. This similarity to organic matter frequently present in tissue samples further limits the use of elemental contrast-based analytical methods, exacerbating the characterization gap.

The pre-treatment techniques used for MNPs samples can significantly impact the detection results. Physical, chemical, and biological methods are currently employed for extracting MNPs, each having its own strengths and limitations. However, finding the right balance between sample complexity and MNPs separation efficiency is challenging. Standardized methods for MNP extraction are not yet widely accepted, and further exploration and comparison is needed to establish best practices. Preserving the integrity of MNPs during extraction is crucial. It is important to avoid using reagents or methods that may damage the MNPs and minimize sample contamination. However, methods that achieve high-efficiency extraction with minimal MNPs loss are currently lacking. Some challenges arise during the screening and counting of MNPs, where particles may be obscured by infiltrating impurities, making accurate counting difficult. Given the diverse chemical, physical, and morphological characteristics of MNPs, there is a broad range of responses in detection methods. Therefore, when assessing the risk of MNPs, ensuring the representativeness and comparability of spectral data is essential. Since different shapes of MNPs have distinct compositions and characteristics, representative spectral composition models must be established for each category of MNPs. Spectroscopic determination methods based on technologies such as Raman and Fourier transform infrared have been widely used, but more comprehensive standard databases need to be developed to enhance the accuracy and reliability of the analyses.

## 4. Future Perspectives

### 4.1. Knowledge Gaps

Despite the summarization of separation and identification techniques in aquatic products, uncertainties persist. A major challenge lies in the fact that MNPs are present in natural aquatic environments in the form of mixtures in which fiber is the main component. Moreover, most laboratory studies have conducted simulated separation experiments using mainly microbeads as a representative of MNPs, which makes most separation methods impractical for practical application. In addition, some researchers have exposed aquatic products to inappropriate environmental concentrations of MNPs, which may not accurately reflect real-world conditions, which lead to laboratory conditions differ significantly from those in the aquatic environment.

Moreover, compared to MNPs, measuring the diameters of NPs can be highly challenging. While there is some understanding of the mechanisms of organic contamination adsorption by MNPs and the hazards of organic contaminants to aquatic products and humans, there is currently a dearth of research on how organic matter enters and adsorbs onto MNPs within human bodies [142]. The process of entry into aquatic products and humans and the changes and residues of these organic contaminants during adsorption are not clear, resulting in a knowledge gap that has implications for the risk assessment of MNPs. It is worth noting that most aquatic toxicity studies of MNPs have been conducted in laboratory settings. However, there remains a critical knowledge gap concerning the ecotoxicological impacts of MNPs on aquatic species and high-trophic-level consumers, including humans.

### 4.2. MNPs for Aquatic Products from a Food Safety Perspective

Over the past decade, considerable efforts have been made to identify MNPs in aquatic products. However, the lack of standardized methods and quality assurance programs presents challenges in interpreting the identification results of MNPs [67]. To address these challenges and improve the efficiency and accuracy of MNP detection, it is essential to establish quality assurance and quality control measures for MNPs. This includes the creation of a global database containing information about detected MNPs, including their size, shape, and material [143,144]. Such a database would be immensely helpful in facilitating research efforts and promoting a unified risk result assessment method for MNPs in aquatic products. By implementing QA/QC measures and collecting global data on MNPs, our understanding of the distribution and risks associated with MNPs in aquatic ecosystems can be enhanced. Therefore, it is necessary to conduct identification experiments with samples of important aquatic products and to discard farmed exposure experiments to achieve more reliable assessments. To ensure a more accurate assessment of MNPs in aquatic environments, the scientific focus should be on increasing the number of laboratory field experiments involving parallel exposures. Thus, leveraging the accumulated experimental experience is a valuable approach to improve the separation and identification of MNPs in ongoing experiments. This iterative process allows researchers to fine-tune their methods and analytical techniques, leading to more accurate and reliable results when analyze MNPs. Moreover, this approach would also deepen our understanding of the actual risks posed by MNPs in natural aquatic ecosystems.

## 5. Conclusions

To address the challenge posed by the presence of MNPs in aquatic products, this review outlines the latest analytical techniques for the separation and identification of MNPs in aquatic organisms, and presents new discoveries concerning the occurrence of MNPs in aquatic products. Furthermore, it delves into the impact of MNPs on aquatic products and human health on the perspective of food safety. In the course of summarizing the presence, effects, and associated risks of MNPs in aquatic products, this review highlights the deficiencies in safety studies and techniques for separation and identification. Indeed, in order to enhance the precision and reliability of analytical results, the future imperative lies in the advancement of standardized methods for the extraction and identification of aquatic MNPs. This not only serves to standardize research related to aquatic MNPs but also allows for a more accurate assessment of MNP hazards.

## Figures and Tables

**Table 1:** MNPs in aquatic products.

Aquatic Products	Region	Main MNPs	Location	Reference
Fish	Mediterranean; New Zealand; Philippines; China coast; India, Bangladesh	Macrofibre, PET, PP, PVC, PP.	Intestine, gills, muscle tissue, eggs, head, stomach	[14,37,38,39,40,41,42,43]
Shrimp	Northern Bangladesh, California, South America, China, India, Atlantic waters	Fiber, filamentary PE, PA, PS, PT, nylon	intestinal glands, stomach, pyloric stomach, gills, exoskeleton	[29,44,45,46,47,48,49,50,51]
Crab	Iran China Zhuhai Musa Bay (aquaculture), Polish Coast, Bering Sea, North Adriatic Coast, Italy, Plumka Island, Indonesia, Kerala Coast, India, Chile	Fiber and fragments of PET, PE, PT, PE, PC, PAM, acrylic	Stomach, digestive tract, foregut and midgut, gills, and muscles	[52,53,54,55,56,57,58]
Bivalves	Qingdao, China, Shanghai Fish Market, Korea, France, Belgium, British coast, Persian Gulf, North Sea (The Netherlands).	Rayon, chlorinated PE, PVC, PVDF, Fiber: PS, nylon	Digestive glands or intestines	[59,60]
Seaweed	Coastal China, USA, Korea	PP, PE, and poly (ethylene propylene) copolymers, fiber	Cells	[61,62]
Mollusks	China, Australia, Norway, and Canada	PE, PES, synthetic cellulose, PVDF, PP, PAN, PA, PC, PU, PS	Digestive glands or intestines	[63,64,65]
Canned sardines, sprats	Morocco, Japan, Iran, Malaysia, Thailand, Vietnam, Germany, Latvia, Poland, Portugal, Scotland, Russia, Canada	PC, PET PP, PE, fiber, film	Muscle tissue, head, stomach	[66]
Dried fish	Malaysia	PVC	Muscle tissue, skin	[67]

**Table 2:** MNP hazards to aquatic products and humanity.

Classification	Category	Hazards	Reference
Humanity	Human gastric cancer cells	Transcription of genes affecting immune function	[73]
	Renal epithelial cells	Endocytosis	[74]
	Human colonic epithelial cells and small intestinal epithelial cells	Intracellular mitochondrial polarization and Rothschild’s enzyme rise	[69]
	Human feces	Nine plastic shapes of MNPs found	[75]
	Human placenta	MNPs detected	[76]
	Human lung tissue	Histopathological changes	[77]
Aquatic products	Pacific oyster	Transcription of genes affecting energy metabolism and development, smaller diameter, and reduced fertility	[78]
	White leg shrimp	DNA damage received	[79]
	Marine mussels	Produces oxidative stress	[80]
	Crustaceans	Neurotoxic effects and oxidative stress	[81]
	Bivalves	Immunomodulation, apoptosis	[82]
	Adult zebrafish	Histopathological changes: tissue changes, neutrophil genesis	[83]
	Mediterranean mussel	Structural changes and necrosis	[84]
	Crucian carp	Brain damage and behavioral disorders in fish	[85]

**Table 3:** Advantages and disadvantages of MNP detection methods.

Testing Methods	Aquatic Organisms	Advantages	Disadvantages	Reference
Visual inspection	Fish, Crab, Bivalves	Simple and easy to handle	Unable to analyze the chemical composition of MNPs	[27,95,104]
SEM, SEM/AFS/EDX, SEM/AFS/FT-TR	Fish, Crab, Bivalves Shrimp	High resolution images High accuracy with simultaneous identification of polymer shape and additive shape Nano analysis, ultra-clear and high magnification images, providing information on elemental composition	Sample coated under high vacuum; no detailed identification information available laborious and expensive sample preparation, no large-area testing, inefficient Higher conditions and larger current laboratory costs	[34,105,106,107]
FT-IR	Bivalves	Non-destructive, perfect, fast, and quite reliable	Less efficient, susceptible to moisture interference, expensive	[108,109,110]
FITR-Raman	Bivalves, Crab	Non-destructive, non-contact analysis; suitable for opaque and dark colored particles	Presence of fluorescent interference and susceptibility to pigment interference	[111]
Raman	Fish	High spatial resolution, a clear advantage in detecting MNPs with particle sizes smaller than 20 um	Weak signals with a low signal-to-noise ratio	[112,113,114]
LC	Crab, and Fish	High recovery rate	Unable to determine physical properties; limitations on the shape of polymer selected	[115,116]
GC-MS	Mussels	Low sample volume required for testing and high accuracy	Complex data Difficult to parse	[67]
TED-GC/MS	Crab	Solvent free; avoids background contamination; sensitive and reliable	A certain weight of pellets per run; the database is only available for PE and PP	[117,118]
Py-GC/MS	Shrimp, Fish	Robust, with relatively short analysis time	For identification of pe and pp only, conclusions are for total mass fraction of participating polymers only; must be combined with concentration methods	[67,119]

## Data Availability

All data analyzed during this study are included in this published article.

## Abbreviations

MPs	Microplastics
NPs	Nanoplastics
MNPs	Micro- and Nanoplastics
BPA	Bisphenol A
SEM-EDX	Scanning Electron Microscopy/Energy-Dispersive X-ray Technique
FT-IR	Fourier Transform Infrared Spectroscopy
Raman	Raman Scattering Spectrometer
FITR-Raman	Fourier Transform Infrared spectroscopy-Raman scattering Spectrometer
LC	Liquid Chromatography
GC-MS	Gas Chromatography-Mass Spectrometry
TED-GC/MS	Thermal Extraction Desorption -Gas Chromatography/Mass Spectrometry
Py-GC/MS	Pyrolysis-Gas Chromatography/Mass Spectrometry
SEM/AFS/EDX	Scanning Electron Microscopy/Atomic Fluorescence Spectrometer/Energy-Dispersive X-ray Technique
SEM/AFS/FT-TR	Scanning Electron Microscopy/Atomic Fluorescence Spectrometry/Fourier Transform Infrared Spectroscopy
POPs	Persistent Organic Pollutants
NOAA	Noaa National Oceanic and Atmospheric Administration
TGA-FTIR-GC/MS	Thermogravimetric Analyzer-Fourier Transform Infrared spectroscopy-Gas Chromatography-Mass Spectrometry
PFAS	Perfluoroalkyl and Polyfluoroalkyl
ATR-FTIR	Attenuated Total Reflection-Fourier Transform Infrared Spectroscopy
QA/QC	Quality Assurance/Quality Control
ROS	Reactive Oxygen Species
